# Nursing students' willingness to work in geriatric care: An integrative review

**DOI:** 10.1002/nop2.726

**Published:** 2020-12-04

**Authors:** Fengling Dai, Yao Liu, Mei Ju, Yufeng Yang

**Affiliations:** ^1^ Faculty of Nursing Southwest Medical University Luzhou China; ^2^ Department of Emergency Medicine The Affiliated Hospital of Southwest Medical University Luzhou China; ^3^ Sichuan Tianyi College Chengdu China

**Keywords:** gerontological nursing, integrative review, nurses, nursing students, willingness to work

## Abstract

**Aims:**

This integrative review aims to explore the willingness of nursing students to work in geriatric care over the past 10 years and to explore the factors influencing nursing students to work in geriatric care.

**Design:**

An integrative review.

**Methods:**

Studies investigating nursing students’ willingness in gerontological nursing work and related influencing factors published in English in Cochrane Library, MEDLINE, Embase, PsycINFO and CINAHL between 2010–2020 were included. Data collected in April 2020.

**Results:**

Twenty‐four studies were analysed. Most studies presented a contradictory or negative attitude about the willingness of nursing students to engage in gerontological nursing work. In most studies that rank the intention to work in nursing fields, gerontological care received the lowest or a relatively low ranking. The main factors affecting work related to gerontological nursing include prior experience caring for older adults, attitudes towards geriatrics, anxiety about ageing, clinical practice environment and living experience with older family members.

## 1 INTRODUCTION

Due to the increase in ageing, meeting the needs of the increasing older population for medical and health services is challenging. According to a WHO survey, by 2050, the proportion of people over 60 years old will increase to 22% (World Health Organization, 2018). There is a large demand for gerontological nurses due to the growth of the ageing population; additionally, the prevalence of chronic and degenerative diseases is high, thus resulting in an unprecedented demand for health care (King et al., 2013), particularly for staff in nursing homes, registered nurses specializing in gerontological care and home care personnel (Carlson & Idvall, 2015).

Although the global ageing population has reached a serious level, encouraging nurses to work in geriatric nursing is challenging in many countries (Neville et al., 2013). Nursing students' expectations regarding gerontological caring employment reflect a low level of aspiration and most students are not intent on entering the long‐term nursing workforce to care for older people (Brown et al., 2008; Happell, 2002; McCann et al., 2010; Neville et al., 2013; Stevens, 2011); this lack of intent of nursing students to work in long‐term nursing is a concern.

Liu et al. (2013) conducted a review of nurses' attitudes towards older people. Neville et al. (2013) explored the reasons why undergraduate nursing students are not choosing gerontology as graduate specialty. Abudu‐Birresborn et al. (2019) examined nurses’ and nursing students’ preparedness to care for older people in lower and middle‐income countries through a scoping review. Algoso et al. (2016) discussed undergraduate nursing students’ attitudes, perceptions and experiences in aged care setting. However, as searched in databases including Cochrane Library, PubMed, Embase, PsycINFO and CINAHL, the evidence about comprehensive review of nursing students' willingness to work in gerontological care and of the influencing factors is limited. In particular, as the ageing population increases, an understanding of nursing students’ desire to work in gerontological nursing and the factors influencing nursing students’ consideration of gerontological nursing work is urgently needed. This understanding can provide guidance for educational and clinical decision‐making and help formulate corresponding measures to encourage more students to engage in geriatric nursing care.

Therefore, this integrative review aims to analyse and criticize the current literature on nursing students' intention towards gerontological nursing work and on factors that influence the willingness of nursing students to pursue careers as gerontological nursing practitioners and to provide relevant evidence regarding this phenomenon.

## 2 METHODS

An integrative review of the literature was conducted using the framework provided by Whittemore and Knafl (2005); this approach allows for the inclusion of diverse methodologies (i.e., experimental and non‐experimental research) and contains five stages, including problem identification, literature search, data evaluation and analysis and presentation. This method can use diverse data sources, thereby developing a holistic understanding of the topic of interest (Hopia et al., 2016).

### 2.1 Literature search

The following databases were searched for articles published between 2010–2020: the Cochrane Library, Medical Literature Analysis and Retrieval System Online (Medline), Excerpta Medica dataBASE (Embase), PsycINFO and Cumulative Index to Nursing and Allied Health Literature (CINAHL). The following three groups of search terms (text words and Medical Subject Headings (MeSH) terms, if available) were used in combination: (a) geriatric nursing, aged care, gerontology nursing, old people, elderly, old age, older adults, older population or elder care; (b) willingness to work, job intention, work, employment intention, employment intent or preference of employment; and (c) nursing students, student nurses or undergraduate student nurses. The reference lists of all included studies were hand‐searched to identify any potentially relevant studies, and the authors were contacted to access additional relevant publications.

### 2.2 Eligibility criteria

All studies investigating nursing students’ attitudes towards gerontological nursing work and related influencing factors and that were published in English were included. To analyse up‐to‐date results on this research topic, only articles published in recent ten years were included. In addition, both qualitative studies and quantitative surveys were included.

### 2.3 Study selection and data extraction

Two reviewers independently assessed the studies for eligibility. After eliminating the duplicates, the studies were first selected based on the title and abstract; then, the full‐text publications were examined. Disagreements were resolved by discussion or referral to a third review author. Any differences were discussed, and agreement among the researchers was achieved. A Preferred Reporting Items for Systematic Reviews and Meta‐Analyses (PRISMA) diagram was presented to outline the screening process used in the literature search.

The data extracted included the author, year, the country where the study was conducted, purpose, data collection and analysis methods, sampling and main results. One researcher extracted all the data from the included studies.

### 2.4 Quality appraisal

The quantitative studies were assessed using the Center for Evidence‐Based Management (CEBM) appraisal of a survey checklist (CEBM, 2014), and the mixed‐methods studies were assessed using the Mixed Methods Appraisal Tool (MMAT; Hong et al., 2018). Two reviewers were independently involved in the appraisal process; disagreements were resolved by discussion or referral to a third review author.

### 2.5 Data analysis

Data analysis was initially undertaken by the primary author and later scrutinized by other authors to ensure accurate interpretation and credibility. As suggested by Whittemore and Knafl (2005), four phases were constituted in this stage: (a) Data reduction. In this phase, the primary sources are divided into subgroups according to the logical system to facilitate analysis; (b) Data display. In this phase, data display matrices and graphs are developed to enhance the visualization of patterns and relationships with and across primary data sources; (c) Data comparison. In this phase, data are iteratively compared with examine data displays to identify patterns, themes or relationships. Specifically, in comparing data, the authors searched for common and unusual patterns, contrasted and compared the patterns and themes, clustered similar themes together and subsumed these themes into more general themes and alternated between the literature and the conclusions drawn to verify the findings and test for plausibility and (d) Drawing conclusions and verification. In this phase, the important elements and conclusions of each subgroup are synthesized into an integrated summation of the phenomenon.

## 3 RESULTS

### 3.1 Search results

**FIGURE 1:**
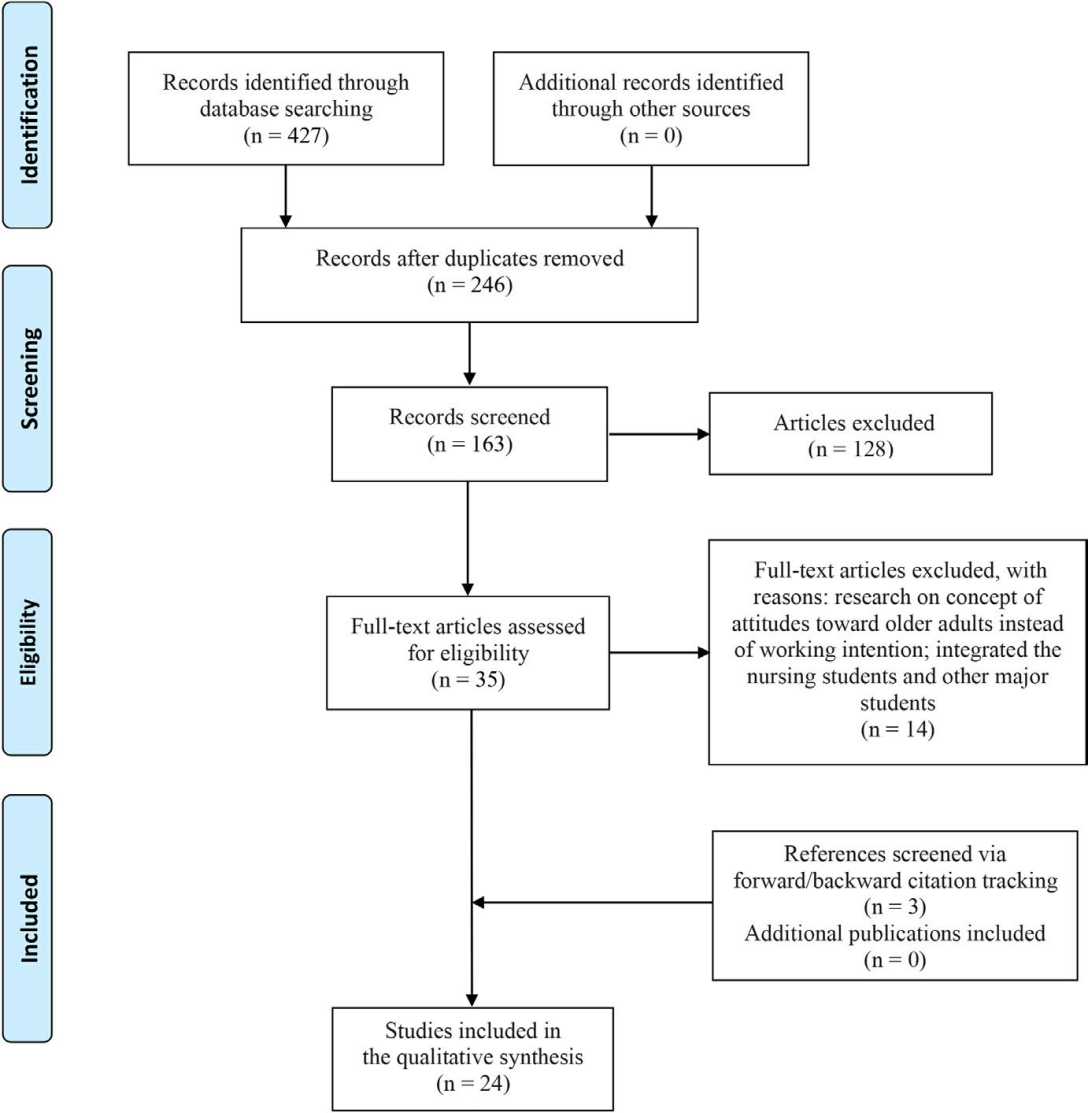
Study selection flow chart

The electronic database search yielded 427 titles and abstracts. After the duplicates were removed, 246 titles and abstracts were screened according to the inclusion and exclusion criteria. This process yielded 35 manuscripts for full‐text review. Fourteen manuscripts were unavailable and were, therefore, excluded. In some studies, the themes focused on students with employment intentions in various departments rather than specifically gerontological care, but these studies also investigated the students’ attitude towards becoming gerontological care practitioners; thus, we included these studies. Overall, 24 publications were included as follows: 19 quantitative publications reporting surveys and five mixed‐method studies; no qualitative study was searched (Figure 1).

### 3.2 Study characteristics

Twenty‐four studies were included in the final review, and the main characteristics of the included studies were extracted (Table 1). Most of the studies were conducted in Australia (*N* = 3), Hongkong (*N* = 1), Israel (*N* = 3), Korea (*N* = 1), Malaysia (*N* = 1), Mainland China (*N* = 3), Sri Lanka (*N* = 1), Sweden (*N* = 1), Taiwan (*N* = 1), Turkey (*N* = 1) and the United States (*N* = 6); additionally, one of the studies was conducted in Australia and China and another one was conducted in both Korea and the United States. Regarding the participant characteristics, overall, 8,892 participants were involved in the study and the average sample size was 370 (range: 50–1,462).

**TABLE 1:** Characteristics of the studies

No.	Reference	Setting	Sample	Research type and method	Instruments	Main results/findings
1	Akpinar Soylemez et al. (2018)	Turkey, faculty of nursing in a university	108 nursing students (46 of the students were educated in an elective geriatric nursing course and 62 were educated in an elective emergency and surgery nursing course)	Quantitative Quasi‐experimental design	Kogan's Attitudes Towards Old People Scale	Students’ willingness to work with an elderly person after graduation was no differences between and both in groups
2	Brabham (2018)	USA: five academic institutions located in the state of Florida	A total of 178 students enrolled in a BSN, ADN and PN programme participated in this dissertation study	Quantitative Non‐experimental Descriptive survey design	Palmore Facts on Aging Quiz 2, Kogan's Attitudes Towards Old People Scale, and the Hartford Geriatric Nurse Competency	Students’ preference to work with older adults postgraduation in the PN group was higher compared with students in both the BSN and ADN group
3	Carlson and Idvall (2015)	Sweden; Malmö University	First‐year student nurses (*N* = 183)	Quantitative A cross‐sectional study	The Swedish version of the Clinical Learning Environment and Nurse Teacher evaluation scale	There were no significant differences between younger students (18–23) and older students (24–50) regarding willingness to work in elderly care or not. Neither was any significant difference displayed between students, based on gender nor for previous work experience
4	Che et al. (2018)	Malaysia: five states in Malaysia	A total of 1,462 nursing students from eleven nursing education institutions	Quantitative A cross‐sectional survey	Intent to Work with Older People Scale and Kogan Attitudes Towards Old People Scale	Malaysian nursing students have a moderate level of intention to work with older people. There were significant differences in effects of gender, ethnic group, academic level, type of nursing institution and setting of older person care clinical experience on intentions to work with older people. There was a moderate and positive relationship between attitudes towards older people and intentions to work with older people, as well as between perceived behavioural control and intentions to work with older people. Attitudes, subjective norms and perceived behavioural control accounted for 19.7% of the variance in intentions to work with older people
5	Cheng et al. (2015)	Mainland China; 7 universities in Shandong	Last‐semester student nurses; *N* = 916 (72 male & 844 female)	Quantitative Cross‐sectional survey	The motivation questionnaire; The Chinese version of the Facts on Aging Quiz I (FAQ I); The Chinese version of the Geriatrics Attitudes Scale (GAS); The gerontological nursing clinical practice environment questionnaire; The Chinese version of the Anxiety about Aging Scale (AAS)	Student nurses' expectancy and value aspects of motivation for choosing gerontological nursing as a career were both at a moderate level; the highest value they held was of personal interest. Clinical practice environment, anxiety about ageing and the attitudes about geriatrics were the main factors influencing student nurses' motivation to choose gerontological nursing as a career in China
6	Cheng et al. (2020)	Hongkong	139 nursing students (69 in SSSP group & 70 in control group)	Quantitative Randomized controlled trial	Kogan Attitudes Towards Old People Scale (KAOP) and a 1‐item scale on Willingness To Care for Older People Scale (WCOP)	No significant difference between the two groups was found. A significant increase of positive attitudes and of willingness to serve older adults was found in both the control group and the group wearing Senior Simulation Suit Programme (SSSP)
7	Chi et al. (2016)	Taiwan; 7 nursing schools in northern, central, southern, and eastern areas of Taiwan	Inclusion criteria: (a) were 20 years of age and older, (b) were enrolled in the school of nursing or department of nursing, and (c) could read Chinese (*N* = 612)	Quantitative A cross‐sectional research	Questionnaire including demographic data, the Attitudes Towards the Elderly Scale, and the Willingness Towards the Elderly Care Scale	Taiwanese undergraduate nursing students had neutral to slightly favourable attitudes towards working with older adults. Nursing students’ positive attitudes about older adults, paying attention to issues related to older adults, and having been a volunteer that served older people were predictors of their willingness to care for older persons
8	Haron et al. (2013)	Israel; Diploma programmes (5 nursing schools) and academic programmes (6 universities and 3 colleges)	*N* = 486	Mixed method Focus groups; A cross‐sectional questionnaire study	A 6‐part structured, self‐administered questionnaire	61% of the 486 respondents had no intention of working in geriatrics, while 12% considered the prospect favourably. 27% of the respondents were prepared to consider geriatric nursing as a career choice only after advanced specialist training in that field. 69% said that the planned expansion of the powers of geriatric nurses would incline them more favourably to work in geriatrics
9	Jang, Oh, et al. (2019)	Korea and United States	437 undergraduate nursing students	Quantitative Cross‐sectional survey design	Scale about frequency and quality of contact with older adults; Anxiety about Aging Scale; Interpersonal Reactivity Index; Semantic Differential Scale; Scale about willingness to care for older adults	Study findings from the entire group showed that nursing students’ willingness to care for the elderly was positively associated with contact quality (*β* = 0.22, *p* < .001) and empathy (*β* = 0.12, *p* = .009) but negatively associated with anxiety about ageing (*β* = −0.23, *p* < .001) and attitude towards the elderly (*β* = −0.14, *p* = .004). Contact quality (*β* = 0.30, *p* < .001) was positively associated with the willingness to care in Korean students, whereas extended family living type (*β* = −0.15, *p* = .012) and attitude towards the elderly (*β* = −0.18, *p* = .005) negatively associated in US students
10	Jang, Kim, et al. (2019)	USA	270 nursing students	Quantitative Descriptive cross‐sectional design	Quality and frequency of contact with older adults; Anxiety of Aging Scale; interpersonal reactivity index; attitude towards older adults; and willingness to care for older adults	The most important factor influencing willingness to care for older adults was the year of the nursing programme (*β* = 0.178, *p* = .003), followed by anxiety of ageing (β = −0.140, *p* = .049) and empathy towards older adults (*β* = 0.13 1, *p* = .031)
11	King et al. (2013)	USA; A large Midwestern University	The first semester (junior year) of the baccalaureate nursing programme(*N* = 80)	Mixed methods A quantitative analysis (questionnaire survey) and a qualitative exploration (focus group)	The Kogan Attitudes Towards Older Adults Scale; and self‐developed by the researchers	Students' attitudes and preference for working with older adults improved over time. However, their preference to work in nursing homes was consistently ranked last among the 10 choices for work preferences. In focus groups, students reported that the gerontological course dispelled myths about caring for older adults, and that clinical placement played a major role in influencing student work preferences
12	Lamet et al. (2011)	USA: A Catholic Southeastern Florida university	Control (*N* = 56) and experimental (*N* = 14) student groups	Quantitative Pretest posttest descriptive cross‐sectional design	Using scales developed by other scholars (Self‐Transcendence Scale and Attitudes Towards Old People Scale)	The CBI improved attitudes towards older people with negative attitudes significantly changed (*p* = .008) but with no significant differences on self‐transcendence and willingness to serve
13	Mattos et al. (2015)	USA; A nursing school in Western Pennsylvania	Quantitative component (*N* = 132): (a) students who completed the gerontological nursing course (*N* = 85); and (b) students who had not yet enrolled in the gerontological nursing course (*N* = 47). Qualitative component: *N* = 72	Mixed methods Paper surveys; semi‐structured interview	Self‐developed by the researchers, and The Facts on Aging Quiz (FAQ‐2), The Geriatric Attitudes Scale (GAS)	Students who were enrolled in the gerontological nursing course or had prior experience with older adults were more likely to report plans to work with this population after graduation
14	McCann et al. (2010)	Australia: a school of nursing in a large Australian city	First year *N* = 88; second year *N* = 45, third year *N* = 95	Quantitative A three‐year longitudinal study	Jorm et al. (1999) “Attitudes and Beliefs about Mental Health Problems: Professional and Public Views” questionnaire	With first‐year students, considerably less were interested in mental health or aged care nursing. By third year, midwifery and aged care were the least preferred careers
15	Natan et al. (2015)	Israel; An academic school of nursing in central Israel	First‐year students (*N* = 200)	Quantitative A cross‐sectional, descriptive design	Kogan's Attitudes Towards Old People Scale	Participants expressed low intention to work in geriatrics upon graduation. Students’ attitudes towards working in geriatrics and normative and control beliefs were found to be predictors of this intention. Additionally, male and religious students were more inclined to work in geriatrics
16	Neville (2016)	Australia; 8 Australian Universities	*N* = 886	Quantitative A cross‐sectional study.	Self‐developed by the researchers based on the Students’ Perceptions of Working with Older People (SPWOP) questionnaire	Australian undergraduate nurses have positive perceptions towards working with older people. However, students did not want to commit to working with older people when they qualified. Factors such as age and gender, which can affect perceptions, were identified
17	Rathnayake et al. (2016)	Sri Lanka; Department of Nursing, University of Peradeniya	First‐ to fourth‐year undergraduate nursing students (*N* = 98)	Quantitative A cross‐sectional study	A self‐administered questionnaire consisting of socio‐demographic variables, Kogan's Attitudes Towards Older People Scale, and questions related to willingness to work with older people	Nursing students have moderately positive attitudes towards older people; however, they show little interest in working with older people. Living with older people develops positive attitudes of young people towards older people. Nursing curricula need to include Gerontological Nursing as a major area
18	Shen et al. (2012)	Mainland China; Chongqing Medical University (CMU)	622 nursing students enrolled in a 4‐year Bachelor of Nursing programme at the university	Quantitative A cross‐sectional survey	Using tool and questionnaire developed by other scholars	Working with older people was ranked as the second to least preferred area by nursing students. Ageist attitudes described as Prejudice was negatively associated with intention to work with older people; while students aged under‐20 were more positively associated with an intention to work with older people
19	Stevens (2011)	Australia: Six campuses within NSW	*N* = 150 (matched over the three administrations)	Mixed methods A replicated longitudinal survey	Using the same survey tool in author's previous research	A career working with older people became less desirable as result of educational processes and experiences within the Bachelor of Nursing programme. In this study, first‐year students entered the programme ranking working with older people 7 out of a possible ten choices. By the end of third year, it was ranked 9th by measuring the mean but had a mode of 10
20	Swanlund and Kujath (2012)	USA; Illinois Wesleyan University	3 first‐year students, 24 s‐year students, 15 third‐year students and 8 fourth‐year students for a total of 50 students	Mixed methods A quantitative study mixed with a qualitative Design	Tuckman–Lorge Attitudes Towards Old People (ATOP) and some opened‐ended questions	The improvement of attitudes towards older adults as students progressed in the programme could be due to exposure to working with older adults in the clinical setting. The choice to work with older adults was based upon experience and time spent with older adults, not attitudes towards older adults
21	Xiao et al. (2013)	Australia and China; One university in Australia and one university in China	3‐year Bachelor of Nursing programme at the Australian university (*N* = 256); 4‐year Bachelor of Nursing programme at the Chinese university (*N* = 204)	Quantitative A cross‐sectional design employed two questionnaires	The 9‐item “Career Choice Questionnaire in Nursing Practice”; The 16‐item “Nursing Students’ Attitudes Towards the Elderly”	The percentage of students more likely to care for the elderly was significantly higher among the Chinese group (72.1%) than the Australian group (45.3%). Work experience with older people and being under the age of 20 were found to be positive predictors, whereas factors such as prejudice towards the elderly and beliefs that elders should live in separate housing were negatively associated with an intention to care for the elderly
22	Yildirim et al. (2011)	Korea; 4 university‐based schools of nursing in Ankara province	447 nursing students. All of the participants in the research were women	Quantitative A cross‐sectional and descriptive study (questionnaire survey)	Self‐developed by the researchers	The lowest percentage wanted to work in psychiatric nursing, geriatrics and care for the handicapped. The primary expectations students had from the workplaces where they wanted to work after graduation been an orientation to the workplace and educational opportunities, opportunities for promotion and job satisfaction
23	Zhang et al. (2016)	Mainland China; Tianjin University of Traditional Chinese Medicine	Participants came from two types of nursing specialty which are common nursing and gerontological nursing (*N* = 382)	Quantitative A cross‐sectional descriptive design.	Care Willingness to the Elderly Scale (CW); Kogan's Attitudes Towards Old People Scale (KAOP); Facts on Aging Quiz (FAQ), and the Gratitude Scale. Structural equation modelling (*SEM*)	For Chinese nursing students, the care willingness of elderly was in medium‐high level. The attitude towards older people, knowledge about ageing, and gratitude were significantly correlated with care willingness. Gratitude plays a mediation role between the knowledge about ageing and care willingness. The experience of caring the elderly could lead to a positive impact in care willingness
24	Zisberg et al. (2015)	Israel; a large academic institution in northern Israel	Attending the 4‐year generic programme for BA and RN degrees (*N* = 224)	Quantitative A cross‐sectional design	Kogan's Old People Scale, Palmore's Facts on Aging Quiz‐1	While knowledge of old age among students increased, preferences for future career in geriatrics declined with education. Ethnicity was a strong predictor of attitudes and future intentions to work with older adults. Culturally tailored educational programmes focused on changing the attitudes towards ageing are critically needed

### 3.3 Study quality

No qualitative research was included according to the criteria after literature searching. No studies were excluded due to poor quality. See Tables 2 and 3 for a summary of the quality assessment.

**TABLE 2:** Methodological quality of quantitative studies

Appraisal questions	Akpinar Soylemez et al. (2018)	Brabham (2018)	Carlson and Idvall (2015)	Che et al. (2018)	Cheng et al. (2015)	Cheng et al. (2020)	Chi et al. (2016)	Jang, Oh, et al. (2019)	Jang, Kim, et al. (2019)	Lamet et al. (2011)	McCann et al. (2010)	Natan et al. (2015)	Neville (2016)	Rathnayake et al. (2016)	Shen and Xiao (2012)	Xiao et al. (2013)	Yildirim et al. (2011)	Zhang et al. (2016)	Zisberg et al. (2015)
1 Did the study address a clearly focused question/issue?	Yes	Yes	Yes	Yes	Yes	Yes	Yes	Yes	Yes	Yes	Yes	Yes	Yes	Yes	Yes	Yes	Yes	Yes	Yes
2 Is the research method (study design) appropriate for answering the research question?	Yes	Yes	Yes	Yes	Yes	Yes	Yes	Yes	Yes	Yes	Yes	Yes	Yes	Yes	Yes	Yes	Yes	Yes	Yes
3 Is the method of selection of the subjects (employees, teams, divisions, organizations) clearly described?	Yes	Yes	Yes	Yes	Can't tell	Yes	Yes	Yes	Can't tell	Yes	Yes	Yes	Yes	Yes	Yes	Yes	Yes	Yes	Yes
4 Could the way the sample was obtained introduce (selection) bias?	Yes	Yes	Yes	Yes	Yes	Yes	Yes	Yes	Yes	Yes	Yes	Yes	Yes	Yes	Yes	Yes	Yes	Yes	Yes
5 Was the sample of subjects representative with regard to the population to which the findings will be referred?	Yes	Yes	No	No	Yes	Yes	Yes	Yes	Yes	No	Yes	No	Yes	No	Yes	Yes	Yes	Yes	Yes
6 Was the sample size based on pre‐study considerations of statistical power?	No	No	No	No	No	No	No	No	No	No	No	No	No	No	No	No	No	No	No
7 Was a satisfactory response rate achieved?	Yes	Yes	Yes	No	Yes	Yes	Yes	Yes	Yes	Can't tell	No	Yes	Can't tell	Yes	Yes	Yes	No	Yes	Yes
8 Are the measurements (questionnaires) likely to be valid and reliable?	Yes	Yes	Yes	Yes	Can't tell	Yes	Yes	Yes	Can't tell	Can't tell	Can't tell	Yes	Yes	Yes	Yes	Yes	Can't tell	Yes	Yes
9 Was the statistical significance assessed?	Yes	Yes	Yes	Yes	Yes	Yes	Yes	Yes	Yes	Yes	Yes	Yes	Yes	Yes	Yes	Yes	Can't tell	Yes	Yes
10 Are confidence intervals given for the main results?	Yes	Yes	No	Yes	No	No	Yes	No	No	No	No	Yes	No	No	Yes	*N*	No	No	No
11 Could there be confounding factors that haven't been accounted for?	Can't tell	Yes	Can't tell	Yes	Yes	No	Yes	Can't tell	Can't tell	Yes	Can't tell	Yes	Yes	Yes	Can't tell	Yes	Can't tell	Yes	Yes
12 Can the results be applied to your organization?	Yes	Yes	Yes	Yes	Yes	Yes	Yes	Yes	Yes	Can't tell	Yes	Yes	Yes	Yes	Yes	Yes	Yes	Yes	Yes

Notes

*Cited from:* Center for Evidence‐Based Management (July 2014), Critical Appraisal Checklist for Cross‐sectional Study. Retrieved (9 June 2019) from https://www.cebma.org.

**TABLE 3:** Methodological quality of mixed‐methods studies

Category of study designs	Methodological quality criteria	Haron et al. (2013)	King et al. (2013)	Mattos et al. (2015)	Stevens (2011)	Swanlund and Kujath (2012)
Screening questions (for all types)	S1. Are there clear research questions?	Yes	Yes	Yes	Yes	Yes
	S2. Do the collected data allow to address the research questions?	Yes	Yes	Yes	Yes	Yes
1. Qualitative	1.1. Is the qualitative approach appropriate to answer the research question?	Yes	Yes	Yes	Yes	Yes
	1.2. Are the qualitative data collection methods adequate to address the research question?	Yes	Yes	Yes	Yes	Yes
	1.3. Are the findings adequately derived from the data?	Can't tell	Yes	Yes	Yes	Yes
	1.4. Is the interpretation of results sufficiently substantiated by data?	Can't tell	Yes	Yes	Yes	Yes
	1.5. Is there coherence between qualitative data sources, collection, analysis and interpretation?	Can't tell	Yes	Yes	Yes	Yes
2. Quantitative randomized controlled trials	2.1. Is randomization appropriately performed?					
	2.2. Are the groups comparable at baseline?					
	2.3. Are there complete outcome data?					
	2.4. Are outcome assessors blinded to the intervention provided?					
	2.5 Did the participants adhere to the assigned intervention?					
3. Quantitative non‐randomized	3.1. Are the participants representative of the target population?					
	3.2. Are measurements appropriate regarding both the outcome and intervention (or exposure)?					
	3.3. Are there complete outcome data?					
	3.4. Are the confounders accounted for in the design and analysis?					
	3.5. During the study period, is the intervention administered (or exposure occurred) as intended?					
4. Quantitative descriptive	4.1. Is the sampling strategy relevant to address the research question?	Yes	Yes	Yes	Yes	Yes
	4.2. Is the sample representative of the target population?	Yes	No	Yes	Yes	No
	4.3. Are the measurements appropriate?	Yes	Yes	Yes	Yes	Yes
	4.4. Is the risk of non‐response bias low?	Yes	No	Yes	Yes	No
	4.5. Is the statistical analysis appropriate to answer the research question?	Yes	Yes	Yes	Yes	Yes
5. Mixed methods	5.1. Is there an adequate rationale for using a mixed‐methods design to address the research question?	Yes	Yes	No	Yes	No
	5.2. Are the different components of the study effectively integrated to answer the research question?	No	Yes	Yes	Yes	Yes
	5.3. Are the outputs of the integration of qualitative and quantitative components adequately interpreted?	No	Yes	Yes	Yes	Yes
	5.4. Are divergences and inconsistencies between quantitative and qualitative results adequately addressed?	No	No	Yes	No	No
	5.5. Do the different components of the study adhere to the quality criteria of each tradition of the methods involved?	No	No	Yes	Yes	No

Notes

*Cited from*: Hong QN, Pluye P, Fàbregues S, Bartlett G, Boardman F, Cargo M, Dagenais P, Gagnon M‐P, Griffiths F, Nicolau B, O’Cathain A, Rousseau M‐C, Vedel I. Mixed Methods Appraisal Tool (MMAT), version 2018. Registration of Copyright (#1148552), Canadian Intellectual Property Office, Industry Canada.

### 3.4 Nursing students' willingness to work in gerontological care

In two studies, the willingness of nursing students to engage in gerontological care was positive (Chi et al., 2016; Zhang et al., 2016). In two other studies, the nursing students' choice of gerontological care as a career and the nursing students’ motivation was at a moderate level (Che et al., 2018; Cheng et al., 2015). However, in two studies, the nursing students had a contradictory attitude. On the one hand, they had a moderate or positive attitude towards older people; on the other hand, the nursing students had no interest in working in gerontological care (Neville, 2016; Rathnayake et al., 2016). In addition, one study showed that the nursing students’ attitudes towards working in geriatrics were negative (Natan et al., 2015). King et al. (2013) found that the negative attitude was related to working in nursing homes rather than to working with older people. In five studies ranking the intention to work in many nursing fields, gerontological care received the lowest or a relative low ranking (King et al., 2013; McCann et al., 2010; Shen & Xiao, 2012; Swanlund & Kujath, 2012; Yildirim et al., 2011). Stevens (2011) indicates that due to the accumulation of the process and experience of nursing education, the willingness to work in gerontological care gradually decreases.

### 3.5 Factors influencing nursing students' willingness to work in geriatric nursing

Twenty‐seven variables were identified from the 24 papers. The relationship between these variables and nursing students' willingness to work in geriatric nursing is summarized in Table 4. The variables were grouped into one of six categories and listed in the order of the most investigated to the least investigated.

**TABLE 4:** Variables related to positive attitudes towards work with older people

Category	Variable	Positive correlation	Negative correlation	Non‐significant correlation
Demographics	Age:younger	Shen and Xiao (2012) and Xiao et al. (2013)	Neville (2016)	Carlson and Idvall (2015) and Che et al. (2018)
	Gender: female	Neville (2016)	Che et al. (2018) and Natan et al. (2015)	Carlson and Idvall (2015) and Mattos et al. (2015)
	Year of study:senior	Jang, Oh, et al. (2019) and Neville (2016)	Che et al. (2018) and Zisberg et al. (2015)	Swanlund and Kujath (2012)
	Religious: yes	Natan et al. (2015)		
	Ethnicity: Arabs	Zisberg et al. (2015)		
Education	Clinical practice environment	Carlson and Idvall (2015), Cheng et al. (2015) and Stevens (2011)		
	Type of nursing institution: public nursing institutions	Che et al. (2018)		
	Type of training programme	Haron et al. (2013): diploma vs. academic		Mattos et al. (2015): traditional BSN VS second‐degree BSN. Che et al. (2018): diploma vs. bachelor. Cheng et al. (2020): Senior Simulation Suit Programme
	Educator: certified in gerontological nursing			Che et al. (2018)
	Gerontological nursing course (vs. integrated into other courses or other courses)	Mattos et al. (2015)		Akpinar Soylemez et al. (2018) and Che et al. (2018)
	Knowledge about ageing	Zhang et al. (2016)		
Experience	Prior experience caring for older people	Cheng et al. (2015), Chi et al. (2016), Haron et al. (2013), Mattos et al. (2015), Neville (2016), Swanlund and Kujath (2012), Xiao et al. (2013), Zhang et al. (2016) and Zisberg et al. (2015)		Carlson and Idvall (2015) and Che et al. (2018)
	Quality of contact	Jang, Oh, et al. (2019)		
Family	Living experience with older family members	Cheng et al. (2015) and Rathnayake et al. (2016)		Che et al. (2018)
	Not the only child at home	Cheng et al. (2015)		
	Parents' attitudes towards older adults was good	Cheng et al. (2015)		
	A close relationship with elderly relatives	Cheng et al. (2015)		
Attitudes	Positive attitude towards the elderly	Che et al. (2018), Cheng et al. (2015), Chi et al. (2016), Haron et al. (2013), Jang, Oh, et al. (2019), Natan et al. (2015), Rathnayake et al. (2016), Zhang et al. (2016) and Zisberg et al. (2015)		Brabham (2018) and Swanlund and Kujath (2012)
	Ageist attitudes (Prejudice, separation)		Shen and Xiao (2012) and Xiao et al. (2013)	
	Anxiety about ageing		Cheng et al. (2015), Jang, Kim, et al. (2019) and Jang, Oh, et al. (2019)	
	empathy	Jang, Kim, et al. (2019) and Jang, Oh, et al. (2019)		
	Gratitude	Zhang et al. (2016)		
Others	Personal interest	Cheng et al. (2015)		
	Expansion of nurse powers in the sector	Haron et al. (2013)		
	Clinical Nurse Specialist role	Haron et al. (2013)		
	Normative beliefs	Natan et al. (2015)		
	Control beliefs	Natan et al. (2015)		

#### 3.5.1 Demographics

Five demographic variables that affect nursing students’ willingness to work in geriatric nursing are mentioned. Three studies showed that younger students were more active in gerontological nursing work (Shen & Xiao, 2012; Xiao et al., 2013). However, Neville (2016) showed that young participants were less active than those in the older age group. Carlson and Idvall (2015) and Che et al. (2018) found the willingness to care for older people did not significantly differ among students of various ages.

There is also a difference in the impact of the gender of nursing students. While two studies showed that males were more likely to work in gerontological care (Che et al., 2018; Natan et al., 2015), another study showed that females were more active than males (Neville, 2016). Moreover, two studies showed no significant correlation between the different sexes (Carlson & Idvall, 2015; Mattos et al., 2015).

Neville (2016) indicated that third‐year participants have more positive perceptions about working with older people. However, Che et al. (2018) and Zisberg et al. (2015) showed the opposite results. Swanlund and Kujath (2012) showed that the willingness to work with elders was not significantly related to the year of study.

Further, religious students were more inclined than secular students to intend to work in geriatrics (Natan et al., 2015). Zisberg et al. (2015) showed that ethnicity was a predictor of intentions to work in geriatric care, and the Arab students demonstrated higher intention to work with older people than Jewish students.

#### 3.5.2 Education

In three studies, an enriched clinical practice environment more positively affected students' selection of gerontological care (Carlson & Idvall, 2015; Cheng et al., 2015; Stevens, 2011).

Haron et al. (2013) showed that the type and place of training made a difference; approximately half the diploma students were prepared to consider working in geriatrics, but only a third of the college students and less than a quarter of the university students were prepared to do so. However, Che et al. (2018) and Mattos et al., (2015) showed that the willingness to work with older people did not differ significantly by the type of training programme.

Mattos et al., (2015) showed that, compared with a gerontological nursing course that was integrated into other nursing courses, a stand‐alone gerontological nursing course yielded students with higher intention levels. Che et al. (2018) showed that the approach used by the gerontological nursing course did not significantly affect the intention to care for older people. In Akpinar Soylemez et al. (2018), no statistical differences were found regarding students’ willingness to work with older people before and after the students’ taking an elective geriatric nursing course and an elective emergency and surgery nursing course. Cheng et al. (2020) evaluated the efficacies of a Senior Simulation Suit Programme; compared with the control group, the programme showed no significant difference.

Moreover, compared with nursing students from public nursing institutions, nursing students from private nursing institutions exhibited slightly lower levels of intention to work with older people. Additionally, the background of nursing educators has no statistical significance (Che et al., 2018). The care willingness towards geriatrics positively correlated with knowledge about ageing (Zhang et al., 2016).

#### 3.5.3 Experience

In our synthesis, nine studies determined that prior experience in caring for older people was positively related to nursing students’ desire to pursue a career in geriatric care after graduation (Cheng et al., 2015; Chi et al., 2016; Haron et al., 2013; Mattos et al., 2015; Neville, 2016; Swanlund & Kujath, 2012; Xiao et al., 2013; Zhang et al., 2016; Zisberg et al., 2015). Nevertheless, Carlson and Idvall (2015) and Che et al. (2018) determined that prior experience in caring for older people did not statistically significantly affect working preference. In Jang et al. (2019), the quality of contact with older people was a positive influencing factor in the willingness of Korean nursing students to care for older people.

#### 3.5.4 Family

Cheng et al. (2015) and Rathnavake et al. (2016) revealed that having a living experience with older family members was a positive factor in geriatric career intention, while Che et al. (2018) showed that there was no significance. Cheng et al. (2015) also indicated that having parents that have good attitudes towards older people and having a close relationship with elder relatives positively affected students’ intention to work with older people.

#### 3.5.5 Attitudes

In our synthesis, nine studies suggested that having a positive attitude towards older people is a positive factor promoting geriatric nursing work among nursing students after graduation (Che et al., 2018; Cheng et al., 2015; Chi et al., 2016; Haron et al., 2013; Jang, Oh, et al., 2019; Natan et al., 2015; Rathnayake et al., 2016; Zhang et al., 2016; Zigberg et al., 2015), while Brabham (2018) and Swanlund and Kujath (2012) found no statistically significant relationship between employment preference to work with older people and students’ attitudes.

Similarly, Shen and Xiao (2012) and Xiao et al. (2013) determined that discriminatory attitudes towards older people were a negatively influencing factor in work related to gerontological nursing. Cheng et al. (2015), Jang et al. (2019) and Jang, Oh, et al. (2019) revealed that anxiety about ageing negatively affects the expectancy and value aspects to choose geriatric nursing as a career and the willingness to care for older people.

In addition, nursing students who had empathy for older people had a high willingness to care for them (Jang, Kim, et al., 2019; Jang, Oh, et al., 2019). Zhang et al. (2016) discovered that gratitude was a mediator between knowledge about gerontological adults and the willingness to care for them.

#### 3.5.6 Others

In our study, we included several influencing factors that are only mentioned in individual studies and cannot be grouped in the above categories.

Cheng et al. (2015) showed that investigators believe that personal interest is an important factor affecting work in gerontological care. The results from Haron et al. (2013) revealed that significantly most of the participants who had planned to consider working in gerontological nursing cited the expansion of the management powers and the creation of the clinical nurse specialist role. Natan et al. (2015) found that normative and control beliefs were predictors of nursing students’ intention to work in geriatrics on graduation.

## 4 DISCUSSION

This study reviewed the willingness of nursing students to work in geriatric nursing care over the past ten years. The results indicated that although some studies showed the willingness of nursing students engaged in gerontological was at a positive or moderate level, more studies presented a contradictory and negative attitude. Furthermore, in most studies where nursing fields were ranked according to the intention to work in these fields, gerontological care was ranked the lowest or ranked relatively low.

A few decades ago, students did not prefer geriatric care. Heller and Walsh (1976) showed that nursing students tend to treat older people with a negative attitude and that negative emotions render these students reluctant to engage in geriatric nursing work; Feldbaum and Feldbaum (1981), Kayser and Minnigerode (1975) demonstrated, in comparing students’ willingness to work in other areas, that most students were unwilling to work in a “nursing home”; Happell’ (1999) also showed that, among students who wanted to work in psychiatry, gerontology was the lowest ranked in terms of willingness to work.

Unfortunately, despite decades of effort, students' willingness to work in gerontological care has not significantly changed or improved. Swanlund and Kujath (2012) suggested that students prefer to work in a fast‐paced working environment, such as acute care departments, rather than gerontological care settings. Compared with paediatric care, intensive care, etc., geriatric nursing is considered to be physically laborious and to have low status and remuneration (Abbey et al., 2006; Neville, 2016; Neville et al., 2008). In an Israeli study, university students' willingness to work in gerontological care was lower than that of college‐ and diploma‐qualified students (Haron et al., 2013) and this finding is similar to the willingness of Chinese nursing students to work in the gerontological care setting. In China, highly educated nursing students are more reluctant to work in a gerontological ward or other institutions care for older people. Hence many institutions care for older people can provide only basic life care, the professional nursing services such as chronic disease management, rehabilitation nursing and palliative care are inadequate. In particular, older people often have multiple chronic diseases, self‐function degradation and decreased self‐care ability; therefore, gerontological care is more complex than simple daily life care and the support and guidance of a more professional and personalized caregiver are needed. Like paediatric and intensive care nursing, the gerontological care specialty requires professional high‐quality nursing personnel.

Regarding the demographic characteristics of the subjects, studies show contradictory results. In terms of age, younger students were more positively engaged than older students in gerontological care work (Shen & Xiao, 2012; Xiao et al., 2013) and senior students hold a negative attitude on geriatric working intention (Che et al., 2018; Zisberg et al., 2015). These findings are inconsistent with the rules of education; it is generally presumed that as the level of students’ education increases, the students’ knowledge of gerontological nursing and willingness towards gerontological nursing work will both improve. The level of engagement should demonstrate an increasing trend.

Concerning gender, female students working in geriatric nursing are more positive (Neville, 2016); however, Che et al. (2018) and Natan et al. (2015) showed the opposite result. Neville (2016) provided an analysis showing that women are more likely to be set up as “role caregivers” in the traditional sense. In an analysis conducted by Natan et al. (2015), the male nurses indicated that they could assist older people with meeting their fitness goals, such as rotation and activities; therefore, gerontological care provided by male nurses is in high demand. In addition, the role of male nurses in traditional women's work is a feature of certain departments, such as maternity wards and finding a related job can be challenging. We believe that the contradictory results obtained by studies conducted in different geographical areas can be explained by cultural differences or may have originated from scientific sources, such as sample size or sample bias due to fewer male nurses.

Concerning educational level, although some researchers have explored various courses and training programmes in recent years, many studies (Akpinar Soylemez et al., 2018; Che et al., 2018; Cheng et al., 2020; Lamet et al., 2011) have failed to find significant differences compared with the control groups or show significant improvements in students’ willingness to work with older people, thus indicating that more efficient educational strategies should be explored.

Geriatric nursing education includes academic and practical clinical training. In some nursing faculties and schools, geriatric nursing courses are available for one semester, mostly at the senior level, and are mainly centred on disease‐centred medical modes (Shen & Xiao, 2012). The distinction between nursing courses on gerontological care and those on general medical care is unclear; the specific characteristics of gerontological nursing are not sufficiently prominent, particularly regarding specialized gerontological nursing skills, such as communication skills, multiple medication nursing and gerontological rehabilitation nursing. Therefore, students who enter clinical practice have difficulty in addressing complex situations in older patients; this difficulty further leads to the negative attitudes held by students towards gerontological care. Besides, Garbrah et al. (2017) presented that nursing curriculums as featuring too much emphasis on acute and critical left nursing students feeling unprepared to work in gerontological nursing, which suggested that adequate gerontology‐related courses should be included in the curriculum for every student irrespective of their specialization option. Concerning curriculum design, nursing educators should incorporate methods to increase interest and promote the attractiveness of lectures so that students will more readily accept geriatric nursing courses. Furthermore, as were evidenced to be the effective learning approaches to improve students’ theoretical knowledge and skills, more education methodologies such as flipped classroom pedagogy, and simulation‐based learning (Hu et al., 2018; Torkshavaned et al., 2020) are encouraged to be explored in designing gerontological nursing programmes.

In clinical training, nursing education regarding gerontological care promotes a positive clinical learning experience that can improve attitudes towards older people and motivate nursing students to prioritize their intentions to engage in gerontological nursing work (Abbey et al., 2006; Brown et al., 2008; Chenoweth et al., 2010; King et al., 2013; Liu et al., 2013; Robinson et al., 2008). Clinical practice includes the clinical ward environment, staff, patients, nurses, teachers and interactions with student tutors (Papp et al., 2002). Schools and hospitals should carefully screen for knowledgeable and caring teachers, train teachers, provide adequate medical supplies and equipment in the internship section and develop a comprehensive internship programme for students (Chi et al., 2016). In particular, regarding the role of teachers during internship, it is important for high‐quality nursing centres to create a harmonious relationship between nurses and patients, provide a good environment and establish a high‐quality nursing service consciousness. As stated by Che et al. (2018), the clinical learning setting is critical for cultivating students’ interest in geriatric care; therefore, nursing programmes should ensure that both the training environment and assigned mentors work to promote positive attitudes towards caring for older people.

In addition, in the past, the treatment and care of the older people were mainly distributed in other disease‐centred specialties departments, not enough attention was paid to the holistic care model regarding older people, thus lead to the development of the geriatrics department of hospitals in many countries was slowly, many people including nursing students often consider gerontology nursing as only working in a nursing home type situation. This reminds educators should give a comprehensive introduction to nursing students about the geriatric care related facilities especially when they are planning their career in geriatric nursing.

Moreover, as prior experience caring for older people positively affects intention to work with them, more activities involving caring for older people are encouraged. Examples of such activities include encouraging older people to participate in community activities; regularly visiting and performing volunteer work for older people in nursing homes; and assisting older people with housekeeping, reading, communicating, etc. Increasing opportunities to interact with older people cultivates patience and responsibility, generally improves knowledge about geriatric nursing and generally develops more experience in working with older people.

Concerning family education and its impact on working intention, the experience of living and interacting with older people in daily life includes both the experience of caring for and the experience of understanding older people. Living with the older members in a family can promote nursing students' understanding of the lifestyle involved with interacting with older people. Compared with students without relevant experience, experienced students have more confidence and skills in caring for older people (Zhang et al., 2016). Students' concern and sympathy for older people can be easily simulated (Pan et al., 2009) and interactions with older people can reduce the anxiety of nursing students regarding ageing (Yan et al., 2011). Furthermore, young persons who live with older people are more likely to be enthusiastic about people who need help because such young persons are more likely to take care of older people (Zhang et al., 2016). The role of parents is vital; parents should be filial to their parents and set a good example for their children to encourage respect for older people.

### 4.1 Limitations

Because of the limitation of language, we included only articles published in English; this restriction may have led to language bias, and some significant findings published in other languages might have been overlooked. Second, although the search strategy was extensive and inclusive, we did not search the unpublished literature, and hence, the related data might be missed. Moreover, the inclusion criteria did not clearly distinguish among gerontological care workplaces, such as geriatrics departments, nursing homes, rehabilitation centres, or general wards at gerontological care pension institutions; the nursing students’ work tendency in different workplaces of geriatric nursing may have differed. Further studies are needed to clarify these issues.

## 5 CONCLUSION

This paper reviewed 24 studies reporting on the willingness of nursing students to work in geriatric nursing over the past ten years and the relevant influencing factors. The results showed that although in recent years, governments, educational systems and professional nursing associations have initiated efforts to promote gerontological care services, nursing students’ willingness to work in gerontological care services is still not promising. And the main factors affecting work related to gerontological nursing include prior experience caring for older adults, attitudes towards geriatrics, anxiety about ageing, clinical practice environment and living experience with older family members.

This finding suggests that continued and dedicated work towards improvements can be achieved by government policies, public opinion, school programmes, clinical practice education, family atmosphere and many other efforts. Given the global ageing population has reached a serious level and the demand for geriatric nurses is expected to increase dramatically, further research on the subject is desirable and timely.

## CONFLICT OF INTEREST

No conflict of interest has been declared by the author(s).

## AUTHOR CONTRIBUTIONS

DF, LY, JM: Study design. DF, YY: Data collection. DF, LY, JM: Data analysis. DF: Manuscript writing. DF, LY: Critical revisions for important intellectual content.

## Data Availability

The data sets generated for this review are available on reasonable request to the corresponding author.
